# Primary Pleomorphic Omental Rhabdomyosarcoma in an Adult: A Report of a Unique Case

**DOI:** 10.7759/cureus.21576

**Published:** 2022-01-24

**Authors:** Ilham A Alteerah, Raouf H Azzuz, Mohamed A Moftah, Yousef M Hasen, Soad I Eldruki

**Affiliations:** 1 Department of Radiology, Benghazi Medical Center, Benghazi, LBY; 2 Department of Medical Oncology, Benghazi Medical Center, Benghazi, LBY; 3 Department of General Surgery, Benghazi Medical Center, Benghazi, LBY; 4 Department of Pathology, School of Medicine, University of Zawia, Zawia, LBY; 5 Department of Pathology, Benghazi Medical Center, Benghazi, LBY

**Keywords:** primary rhabdomyosarcoma, radiotherapy, chemotherapy, omentum, pleomorphic rhabdomyosarcoma

## Abstract

Rhabdomyosarcoma (RMS) is a very rare, highly malignant neoplasm thought to originate from the pluripotent mesenchymal tissue. Predominantly diagnosed among children and teenagers, however they can also be encountered in adults. There are a few risk factors associated with RMS like family history of malignancy and genetic syndromes like neurofibromatosis type 1, Li-Fraumeni syndrome, Noonan syndrome and Beckwith-Wiedemann syndrome; however, most cases of RMS are sporadic. Other factors like radiotherapy for other malignancy and pre-natal radiation exposure also are associated with increased risk of developing RMS. The most common reported sites for RMS are head, neck, trunk, pelvis and lower limbs. Omental involvement of primary RMS has been rarely reported in the literature. Principally, the survival of treated RMS cases has improved, primarily due to multidisciplinary management approaches. In this paper, we report a case of primary pleomorphic RMS in a 50-year-old female who presented with abdominal pain.

## Introduction

The tumour nomenclature is derived from the Greek words rhabdo, which means rod shape, and myo, which means muscle. Rhabdomyosarcoma (RMS) is recognised as the most frequent childhood soft-tissue sarcomas, and it is encountered in more than 50% of all soft-tissue sarcomas in the paediatric and adolescent age groups [1]. It is less frequently encountered in the adult population, and in the published literature, particularly, the primary omental RMS has been rarely reported [2,3]. Diagnosis of such omental tumours can be delayed, as its initial symptoms are non-specific. The presenting symptoms of abdominal tumours may include abdominal discomfort, pain, distention, mass and constipation [2,4,5]. According to the World Health Organisation (WHO), pleomorphic rhabdomyosarcoma (PRMS) is a morphological variant of RMS that typically occurs in adults. The molecular genetic anomalies in PRMS are not yet completely explored and understood [6]. Furthermore, to the best of our knowledge, there are no reported cases of omental PRMS that have been published in the literature. Hereby, we report a case of primary omental PRMS in a 50-year-old female.

## Case presentation

**Figure 1:**
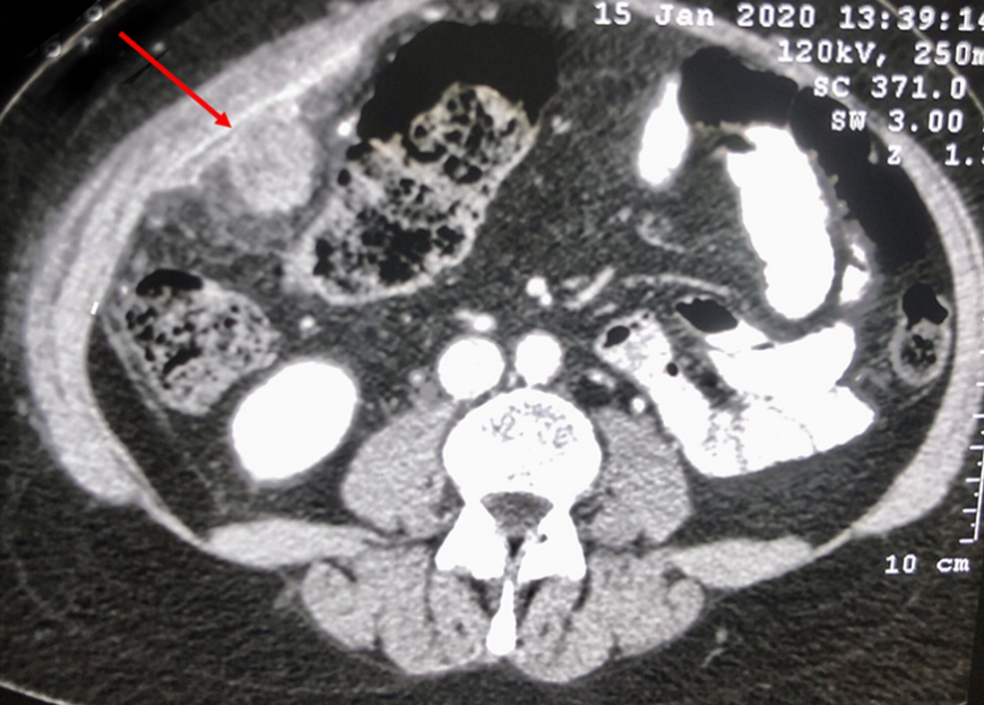
Computed tomography scans with contrast enhancement demonstrating the ill-defined enhanced soft tissue tumour noted posterior to the right anterior abdominal wall (red arrow indicates tumour).

A 50-year-old female Libyan patient presented with a recent history of abdominal pain associated with vomiting and constipation. Two years prior to the presentation, she was diagnosed with ovarian papillary serous cyst adenocarcinoma FIGO stage II C (International Federation of Gynecology and Obstetrics Classification) treated with total abdominal hysterectomy, bilateral salpingo-oophorectomy and inguinal lymph node dissection in addition to six cycles of chemotherapy (paclitaxol, carboplatin, avastin and gemzar). Clinically, there was a tender mass felt at the right lower abdominal quadrant close to umbilicus area. Laboratory findings showed that haemoglobin level was 7.7 g/dL, and other results were not remarkable. A computed tomography (CT) scan revealed a 6.5 × 6.3 × 5.5 cm ill-defined enhanced soft-tissue lesion posterior to the right anterior abdominal wall muscles, corresponding to L2-L4 level. The right-side abdominal wall muscle was thickened and surrounded by omental fatty stranding. There was no clear cleavage between the mass and the anterior abdominal wall as shown in Figure 1.

**Figure 2:**
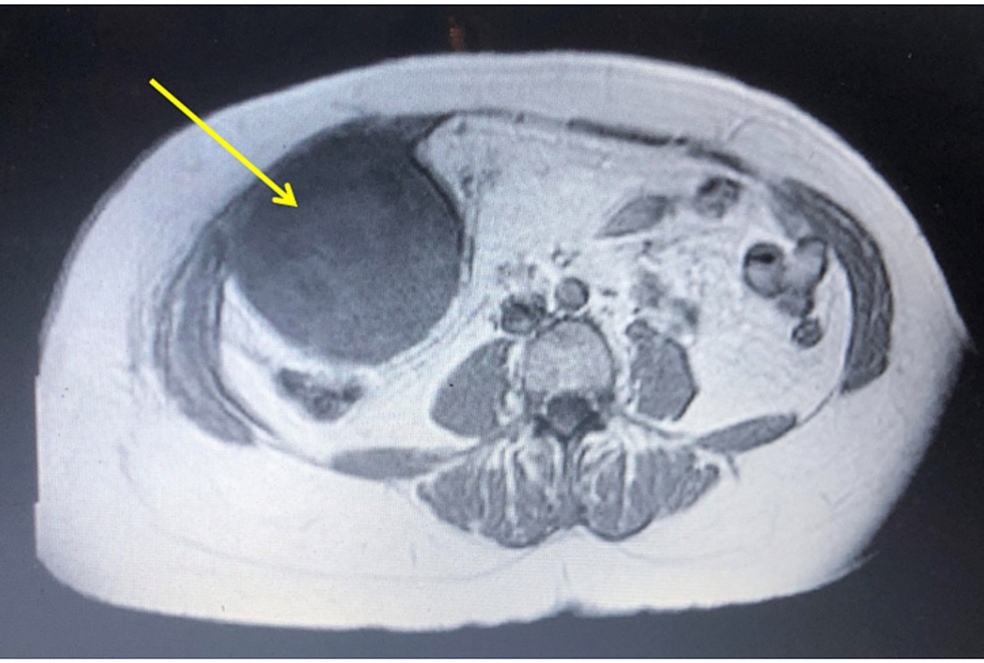
: Magnetic resonance imaging abdomen with contrast enhancement demonstrating the ill-defined enhanced soft tissue tumour noted posterior to the right anterior abdominal wall (yellow arrow indicates tumour).

These findings were confirmed by a magnetic resonance imaging (MRI) study, which showed a well-capsulated non-enhancing heterogeneous intraabdominal extraperitoneal lesion seen adherent to the posterior aspect of the right transversus abdominis muscle as shown in Figure 2.

**Figure 3:**
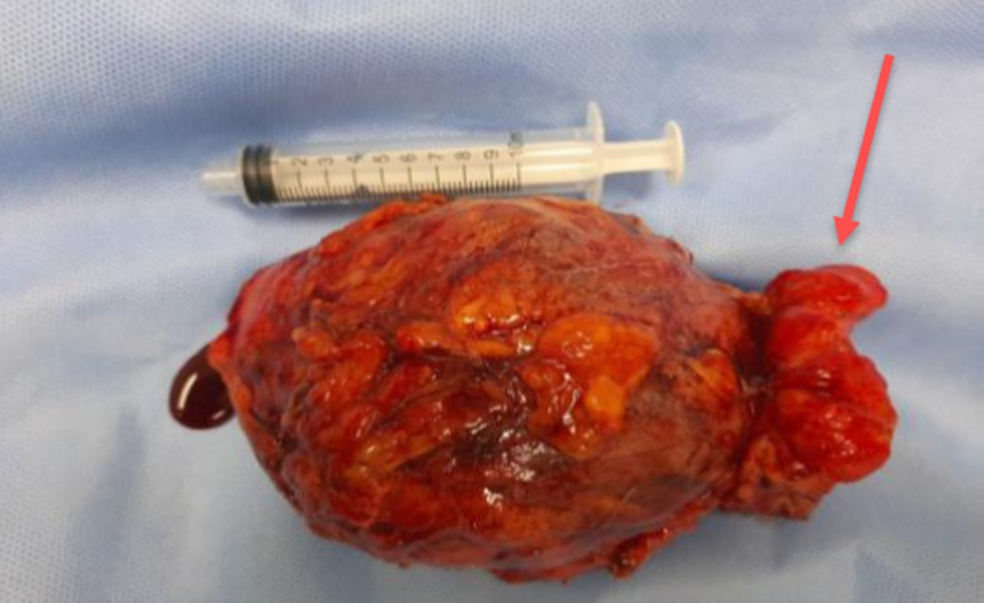
Macroscopic view of the excised anterior abdominal wall tumour measuring 11 × 9 × 7 cm with segment of ascending colon (red arrow points to a resected segment of ascending colon).

Initially, it was assumed that the mass was a malignant tumour due to ovarian cancer recurrence to the omentum or retroperitoneum. Imaging-guided core biopsy was performed; however, the sample was inadequate. The patient declined a repeat attempt and her choice was to go straight for surgery, and the staging CT chest and pelvis did not reveal any additional disease foci. The patient underwent an exploratory laparotomy, which revealed a large right-sided abdominal mass about 10 × 10 cm in size involving the omentum; therefore, it was completely resected with part of the anterior abdominal wall muscle and ascending colon that was adherent to the tumour. Gross examination showed that the resected firm tissue mass measured 11 × 9 × 7 cm with central necrosis and haemorrhage as shown in Figure 3.

**Figure 4:**
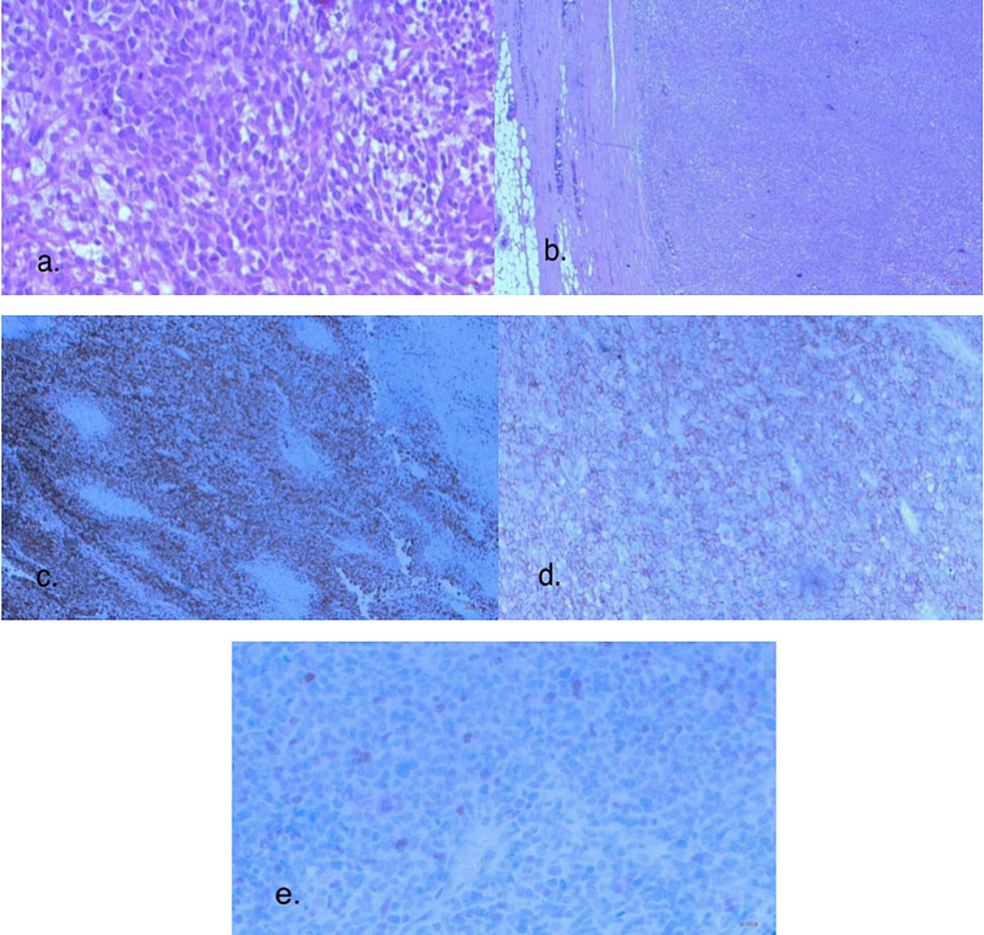
(a, b) H&E: Fragments of high-grade malignant mesenchymal tumour composed of densely cellular fascicles of spindle cells and tumour cells are seen. (c) Desmin positive expression. (d) CD56 positive expression. (e) Myogenin positive expression. H&E: haemotoxylin and eosin.

Microscopically, it showed fragments of high-grade malignant mesenchymal tumour composed of densely cellular fascicles of spindle cells alternating with hypocellular myxoid areas creating marble-like appearance and admixed with sheet of round polygonal cells with densely eosinophilic cytoplasm associated with wide areas of tumour necrosis. The malignant cells display moderate to marked nuclear pleomorphism and high mitotic figures. Many large bizarre tumour cells are seen. The tumour cells are seen invading the adipose tissue and the serosal surface of the large bowel wall as shown in Figure 4. The immuno-histochemistry (IHC) study was done and showed that the malignant cells were positive for vimentin, desmin, myogenin and CD56 (neural cell adhesion molecule) as shown in Figure 4. The later marker was tested in this case as it is frequently expressed by alveolar RMS tumour cells; however, it is not specific. Further IHC assays revealed that the tumour is negative for PAN-CK (pan-cytokeratin), LCA (leukocyte common antigen), S100, CD34, inhibin, EMA (epithelial membrane antigen) and SMA (smooth muscle actin). The final diagnosis of tumour was consistent with completely excised high-grade PRMS.

Postoperatively, the patient recovered uneventfully and resumed her daily activities few days after surgery and the patient was referred to the oncologist for further management. Follow-up MRI abdomen was performed 7 and 10 months after the surgery and revealed no evidence of disease recurrence.

## Discussion

The omentum may harbour a metastatic tumour from extra/intra-abdominal primary tumours but, on the other hand, primary tumours originating from the omentum may be encountered; however, they are very rare. RMS is a very rare, highly malignant neoplasm thought to arise in immature striated skeletal muscle cells at any age and any site of the body. Generally, RMS is the most often encountered subtype of soft-tissue sarcomas, and it only accounts for 3% of childhood cancers and 2% of adolescent cancers [1]. It is extremely infrequent in adults and it accounts for less than 1% of all adult cancers [2]. The reported sites for primary RMS are head, neck, upper limbs [7], legs, trunk, pelvis [8], uterus [9-11], liver [5,12], ovary [13] and mesentery [14]. The omental tissue is of mesothelial origin, and it possesses stem cells' function, and angiogenic, immune and fibrotic activities. All these elements are crucial in the omental functions like promoting vascularisation, limiting intra-peritoneal infection, securing viscus perforation sites and healing acceleration. These listed factors may take part in the pathogenesis of primary omental tumour formation and metastasis. The intraperitoneal involvement of RMS is uncommon and rarely affects adults. The patients of the adult age group with RMS had worse survival outcome than children with similar tumours [15]. After extensive literature review, only two cases of RMS with omental involvement have been reported yet. The common presenting symptoms of omental tumors are not specific; however, they include abdominal discomfort (45.5%), abdominal mass (34.9%) and abdominal distention (15.2%) [16]. Our case presented with abdominal pain, which is similar to the case that was reported by Pathak P et al. (2015), in which they had presented a case of a 21-year-old man with abdominal pain and a lump in the left hypochondrial region [4]. On the other hand, Seenu et al. (1995) published a case of a 45-year-old male who presented with pyrexia [17]. According to the 4th edition of the WHO Classification of Tumors of Soft tissue and Bone, there are four main histological types of RMSs. These include embryonal, alveolar, pleomorphic, and undifferentiated or anaplastic [18]. Moreover, Sultan I, et al.'s study showed that PRMS was rarely seen in patients younger than 20 years. In addition, the prognosis for adults with this subtype was significantly worse than for patients with other subtypes, including alveolar RMS [15]. Radiologically, in our case, there are no specific features that differentiate the nature of the mass or localise its primary origin, and this is probably due to the extent of the omental disease and its adherence to the transverse colon. The other diagnostic challenge in this case was the differential diagnosis of metastatic tumour from the previously treated ovarian malignancy or a new primary metachronous omental tumour; surgical exploration with complete resection of the omental RMS was performed successfully. The diagnosis of PRMS was made based on the typical cellular morphology of high-grade malignant spindle cells that display skeletal muscle differentiation, and IHC stains. The tumour cells express desmin, CD56, myogenin and vimentin, but not PAN-CK, LCA, S100, CD34, inhibin, EMA and SMA. Due to non-availability, we were unable to screen the tumour for CTNNB1 gene, and this alteration is diagnostic for other tumours as desmoid fibromatosis, which is an important differential diagnosis in this case. Some authors recommend another useful biological marker, the Ki-67 proliferation index, and it is a measure of cellular proliferation that is commonly used as a significant prognostic indicator in breast and colon cancers, soft-tissue sarcomas, as well as RMS [19,20].

The current treatment of RMS is multifaceted and comprises surgical excision, chemotherapy and radiotherapy. PRMS is treated like soft-tissue sarcoma using a multidrug chemotherapy protocol that includes ifosfamide, doxorubicin and mesna. Regarding the radiotherapy, the proton beam therapy is the standard type of radiotherapy for most of the RMS cases. The targeted RMS therapy is increasingly being incorporated into clinical practice, as IGF1R (insulin-like growth factor 1 receptor)-directed targeted therapy has revolutionised RMS tumour treatment; however, further development is still required in this field. Some authors claimed that patients with localised RMS can be cured or in remission for a long period, and the outcomes in those with metastatic or recurrent RMS remain poor. Generally speaking, and due to the inefficiency of the adjuvant therapy, the outcome remains poor in the majority of the reported cases [11]. Also, the published reports mentioned that all primary sites of RMS showed a poorer five-year disease-specific survival for adults when compared to the paediatric community [9].

## Conclusions

In this case we have described what we believe to be the third case of primary omental RMS in an adult to be reported in the literature, and the first case ever reported of PRMS of the omentum in adults or children. RMS should be considered in the differential diagnosis of a malignant omental mass in an adult, especially in the absence of another primary site of the tumour, and this is to ensure early diagnosis and appropriate management. PRMS rarity results in the fact that the available information regarding the disease's clinical and biological characteristics are very limited; further multi-institutional trials and researches that are more challenging, however, are still required. 
